# Phosphatidylserine-Dependent Clearance of Damaged Red Blood Cells by Liver Sinusoidal Endothelial Cells in Alcohol-Related Liver Disease

**DOI:** 10.3390/biology15090699

**Published:** 2026-04-29

**Authors:** Siyuan Li, Chaowen Zheng, Xiaowei Zha, Johannes Mueller, Anne Dropmann, Seddik Hammad, Steven Dooley, Sebastian Mueller

**Affiliations:** 1 Center for Alcohol Research, University of Heidelberg, 69120 Heidelberg, Germany; siyuanli.med@gmail.com (S.L.); chaowenzheng5@gmail.com (C.Z.); joh-mueller@gmx.de (J.M.); 2 Department of Neurosurgery, Heidelberg University Hospital, 69120 Heidelberg, Germany; xiaowei.zha@stud.uni-heidelberg.de; 3 Molecular Hepatology Section, Medical Faculty Mannheim, Heidelberg University, 68167 Mannheim, Germany; anne.dropmann@medma.uni-heidelberg.de (A.D.); seddik.hammad@medma.uni-heidelberg.de (S.H.); steven.dooley@medma.uni-heidelberg.de (S.D.); 4 Department of Forensic Medicine and Veterinary Toxicology, Faculty of Veterinary Medicine, South Valley University, Quena 83523, Egypt

**Keywords:** alcohol-related liver disease, efferocytosis, eryptosis, liver sinusoidal endothelial cells, red blood cells, heme oxygenase-1, hemolysis, iron regulation, stabilin-1, CD206, phosphatidylserine, iron metabolism

## Abstract

Alcohol-related liver disease (ALD) and ALD-related mortality are associated with hemolysis, increased erythrophagocytosis, and disturbed iron homeostasis. While macrophage-mediated erythrophagocytosis is well established, we investigated the contribution of liver sinusoidal endothelial cells (LSECs) to handling oxidatively damaged or ethanol-primed red blood cells (RBCs) in ALD. Live-cell imaging demonstrated that damaged RBCs were rapidly taken up by SK-HEP1 cells, an endothelial cell line with LSEC-like characteristics, and RBC uptake was associated with induction of heme oxygenase-1 (HO-1) and activation of its upstream regulator Nrf2. siRNA-mediated knockdown of the scavenger receptor Stabilin-1 attenuated RBC-induced HO-1 expression, supporting a role for Stabilin-1 in efferocytic signaling. Exposure of RBCs to ethanol concentrations as low as 25 mM induced phosphatidylserine externalization and rendered erythrocytes efferocytosis-competent. Lysed RBCs and free hemin elicited comparable oxidative stress responses. In murine models of hemolysis and chronic ethanol feeding, hemoglobin-derived signals were detected within sinusoidal structures showing a diffuse CD206-positive distribution pattern consistent with the sinusoidal scavenger compartment. Similar signals were observed in sinusoidal endothelial regions in human heavy drinkers with clinical signs of hemolysis. Together, these data suggest that LSECs may represent an additional component of RBC clearance in ALD, alongside macrophages and hepatocytes, with implications for hepatic iron handling.

## Simple Summary

Alcohol-related liver disease is associated with the increased destruction of red blood cells. These damaged cells must be removed efficiently because the release of hemoglobin and iron can harm the liver. While this clearance process is mainly known to occur in liver macrophages, the possible role of liver blood vessel cells has remained unclear. In this study, we investigated whether specialized endothelial-like cells that line the small blood vessels of the liver also participate in removing damaged red blood cells. Using live-cell imaging, cell culture experiments, mouse models, and liver samples from patients with alcohol-related liver disease, we found that these endothelial-like cells can take up damaged red blood cells. We further identified the surface receptor stabilin-1 appears to contribute to this uptake process. In addition, we observed that even relatively low levels of alcohol can damage red blood cells and mark them for removal by liver cells. Our findings suggest that endothelial cells of the liver represent an additional component of the system that clears damaged red blood cells, alongside macrophages and liver cells. This process may influence how iron accumulates in the liver during alcohol-related liver disease.

## 1. Introduction

Alcohol-related liver disease (ALD) remains the most frequent cause of liver cirrhosis in Europe [1,2]. About one-quarter of global cirrhosis-related deaths are attributable to alcohol consumption [3]. Most patients with ALD are diagnosed only at the decompensated stage [4], often after years of subclinical progression. Laboratory features commonly include elevated levels of AST, ALT, γ-GT, ferritin, and mean corpuscular volume (MCV) [5]. Non-invasive assessment of fibrosis by transient elastography has emerged as standard diagnostic tool [5,6]. However, due to variable clinical presentation and the lack of specific biomarkers for alcohol intake, ALD often remains underdiagnosed. In a prospective 15-year follow-up of heavy drinkers, we recently identified hemolysis as a key predictor of mortality in ALD [2,7]. In the same cohort, pathological iron overload was observed in nearly 50% of liver biopsies, affecting both hepatocytes and Kupffer cells [7]. This iron accumulation has been linked to disrupted erythrocyte turnover and increased hepatic erythrophagocytosis [8]. Under physiological conditions, senescent red blood cells (RBCs) are phagocytosed primarily by macrophages, allowing efficient iron recycling [9,10]. In ALD, however, this homeostatic process appears to be dysregulated. Hemolysis is highly toxic due to the release of hemoglobin and free heme, a potent pro-oxidant and pro-inflammatory compound [11]. To mitigate this toxicity, damaged or ruptured RBCs must be efficiently cleared. The primary mechanism for this is normally erythrophagocytosis by macrophages, as mentioned above, followed by degradation of heme through heme oxygenase (HO), encoded by the HMOX gene, which produces carbon monoxide (CO), biliverdin, and iron [11,12,13,14]. The iron is stored in ferritin within hepatocytes and macrophages, with ~90% of total body iron recycled from senescent RBCs [9,10,15]. Biliverdin is further reduced to bilirubin [16]. Of the two HO isoforms, HO-1 is stress-inducible and plays a key role in protecting against heme toxicity [14,17,18,19], regulated in part by the Keap1–Nrf2–Bach1 axis [20,21]. In addition to direct phagocytosis of senescent RBCs, macrophages also remove free hemoglobin and heme via distinct scavenger systems: hemoglobin binds to haptoglobin (Hp) and heme to hemopexin (Hpx), forming complexes internalized by CD163 and CD91, respectively [22,23]. CD163 is expressed on tissue macrophages [24] and confers protection against hemoglobin-induced oxidative stress [25]. Soluble CD163 (sCD163) serves as a clinical marker of macrophage activation and correlates with hepatic inflammation and fibrosis in ALD [26,27,28]. We previously showed that heavy alcohol use promotes erythrocyte membrane damage and hemolysis, leading to enhanced uptake of RBCs by hepatic macrophages through CD163—mediated pathways [8]. This was accompanied by robust induction of HO-1 and Nrf2, indicating activation of the cellular defense machinery. In addition, recent work has demonstrated that oxidatively damaged and ethanol-primed red blood cells can be internalized not only by macrophages but also by hepatocytes [29], indicating that erythrocyte clearance in alcohol-related liver disease involves multiple hepatic cell types. Thus, while liver-resident macrophages and hepatocytes are known players in removing red blood cells in ALD [8,29,30], the potential contribution of liver sinusoidal endothelial cells (LSECs) to RBC clearance has not been fully addressed. Liver sinusoidal endothelial cells represent a specialized scavenger interface at the blood–liver barrier and express multiple endocytic and lectin receptors, including Stabilin-1 and the mannose receptor CD206. These cells display a characteristic continuous sinusoidal distribution pattern that is anatomically distinct from the punctate, cell-restricted staining pattern of Kupffer cells and accumulating evidence suggests that red blood cell handling in the liver is not restricted to a single cell type. Prior reports suggest that LSECs may tether damaged RBCs within the hepatic microvasculature [31,32], and iron accumulation in LSECs has been documented in sickle cell disease [33]. Other endothelial cell lines, including HUVECs, have demonstrated phosphatidylserine (PS)-dependent uptake of RBCs [34,35], but mechanistic studies on LSECs remain sparse. LSECs not only participate in local erythrocyte sequestration but are also central regulators of systemic iron homeostasis. Recent work from our group demonstrated that LSEC-derived bone morphogenetic protein 6 (BMP6) is a key inducer of hepatocellular hepcidin expression via SMAD signaling in an endothelial–hepatocyte coculture systems [36]. This BMP6-driven hepcidin induction was highly responsive to both ferric iron and heme, supporting a model in which LSECs integrate local iron signals and translate them into systemic hepcidin responses. These findings highlight the broader functional repertoire of LSECs in orchestrating iron metabolism and suggest a potential mechanistic link to iron overload in ALD. In this study, we investigated whether liver sinusoidal endothelial cells can participate in the clearance of oxidatively damaged or ethanol-primed red blood cells in alcohol-related liver disease. Using complementary in vitro, in vivo, and human tissue approaches, we examined the capacity of LSEC-like tumor-derived cells to internalize red blood cells and to engage stress-responsive pathways linked to heme and iron handling. Particular attention was paid to the potential involvement of Stabilin-1 and downstream induction of HO-1 and Nrf2 signaling. Together, these data indicate that red blood cell clearance in alcohol-related liver disease is a multicellular process involving macrophages, hepatocytes, and liver sinusoidal endothelial cells, with implications for hepatic iron handling and homeostasis.

## 2. Materials and Methods

### 2.1. Co-Culture, Treatments, and In Vitro Efferocytosis Model

SK-HEP1 cells, a cell line derived from a male patient with hepatic adenocarcinoma [37,38] with endothelial-like features and surrogate for LSECs, were cultured with low-glucose Dulbecco’s Modified Eagle Medium (DMEM, Sigma-Aldrich, Taufkirchen, Germany) and supplemented with 10% fetal calf serum (FCS). The conditions were maintained in an atmosphere containing 21% oxygen and 5% CO_2_. RBCs were isolated from fresh human EDTA blood samples. A direct co-culture system of SK HEP1 and RBC together was established by seeding SK-HEP1 at 2 × 10^5^ cells/well in 12-well plates. Then, different doses of RBCs were added to the wells 24 h later. RBCs were either oxidized using CuSO_4_ (0.2 mM) and Ascorbate (5 mM) for 2 h and then washed with 0.9% sodium chloride solution or primed for efferocytosis by different concentrations of ethanol (up to 1000 mM). These oxidized RBCs are used as surrogates for senescent or oxidatively damaged RBCs. The ethanol concentrations were chosen to model a broad range of exposure conditions, including systemic and transient local levels during alcohol intake, rather than steady-state blood alcohol concentrations. Different concentrations of oxiRBC were obtained by diluting oxiRBC in 0.9% sodium chloride solution [8]. To prepare lysed RBCs, 10 mL of 1X RBC Lysis Buffer (Invitrogen, Carlsbad, CA, USA, 00-4333-57) was added per 1 mL of human blood and incubated for 10 min at room temperature and centrifuged at 500× *g* for 5 min at room temperature. Afterwards, the supernatant was decanted and the pellet was resuspended in the 0.9% sodium chloride solution.

### 2.2. Chemicals and Reagents

The following reagents were used in this study: copper-(II)-sulfate (CuSO_4_, Cat. no.: 451657), ethanol, hemin (Cat. no.: 51280) and (+)-sodium-L-ascorbate (Cat. no.: 134-03-2) were purchased from Sigma-Aldrich (Taufkirchen, Germany).

### 2.3. Life Video Camera System

Live videos of the uptake of RBCs by SK-HEP1 cells were taken using the IncuCyte^®^S3 system (Essen BioScience, Royston, UK; Cat. no.: 4647). This live-cell imaging platform allows real-time acquisition of well-wide cell images and videos. Images were captured every 2 min, which is the minimum time resolution supported by the device.

### 2.4. siRNA Transfection

Stabilin-1 siRNA transfection assay was performed on SK-HEP1. Cells were seeded at 2 × 10^5^ per well in 12-well plates, reaching 80–90% confluence. Transfection was conducted in reduced serum medium (Thermo Fisher, Waltham, MA, USA; 31985062) using 30 nM Stabilin-1 siRNA (Santa Cruz Biotechnology, Dallas, TX, USA; sc-45785) or 50 nM non-targeting siRNA (Thermo Fisher; SIC001) as a negative control, transfected with Lipofectamine 2000 (Invitrogen; 13778030) at 3 μL per well. Transfection reagents were removed after 6 h, and efficiency was assessed at 48 h. After 24 h of transfection, cell confluence was approximately 50–60%. Knockdown efficiency was validated using real-time quantitative PCR (qRT-PCR).

### 2.5. qRT-PCR and Western Blotting

Total RNA from cells was extracted using Trizol Reagent (Thermo Fisher Scientific, Waltham, MA, USA; 15596018) following the standard protocol provided by the supplier. PCR was performed by the SYBR green method. Primers (see Supplementary Table S1A) were synthesized at Eurofins Genomics Germany GmbH (Ebersbach, Germany). Real-time quantitative PCR (qRT-PCR) was performed and read by the LightCycler PCR system (Roche Diagnostics, Mannheim, Germany). Results were analyzed using the double delta Ct method [39]. Cells were washed in ice-cold 1 × PBS and harvested in RIPA buffer plus 1 × Complete^®^ protease inhibitor with EDTA (Roche Applied Sciences, Mannheim, Germany) on ice. Equal protein loading was confirmed by protein staining with Ponceau-S solution as well as β-actin/GAPDH. Primary and secondary antibodies are listed in Supplementary Table S1B. Immune-reactive bands were detected by the Odyssey CLx imaging system. Band intensities were quantified using ImageJ (version 1.54d, NIH, Bethesda, MD, USA) for further statistical analysis.

### 2.6. Cell Viability Assay

An MTT assay was conducted to evaluate cell viability as described previously [36]. SK-HEP1 cells were seeded in 96-well plates at a density of 4 × 10^4^ cells per well and incubated overnight. In the following, cells were treated with varying concentrations of oxiRBC with or without a 24-h recovery period, 20 µL of MTT solution (5 mg/mL in PBS) was added to each well. The plates were then incubated at 37 °C for 4 h. After incubation, the resulting formazan crystals were dissolved in 150 µL of dimethyl sulfoxide (DMSO), and the absorbance was measured at 570 nm using a microplate reader (FLUOstar Optima, BMG Labtech, Ortenberg, Germany).

### 2.7. Animal Model of Chronic Alcohol and Hemolysis

We used a mixed acute and chronic alcohol exposure mouse model to study hemolysis [40]. Male C57BL/6 strain mice aged eight weeks were sourced from Charles River Laboratories and housed individually in ventilated cages under sterile, pathogen-controlled environments with 12-h light/dark cycles. This protocol received authorization from the Baden-Wuerttemberg Animal Ethics Committee Karlsruhe (G155/15). For the control group (n = 6), wild-type C57BL/6J mice underwent unrestricted consumption of a Lieber–DeCarli control diet (F1259SP, Bio-Serv, Frenchtown, NJ, USA) for 3 weeks with restricted access to standard chow and water to facilitate adaptation to liquid feeding. The long-term ethanol-exposed group (n = 6) received a Lieber–DeCarli 5% *v*/*v* ethanol diet (F16797SP, Bio-Serv, Frenchtown, NJ, USA) continuously for 3 weeks with an additional two intragastric gavages of alcohol (31.5% wt/vol) within a 24 h interval. Control mice were administered dual isocaloric maltose dextrin boluses (45% wt/vol) at 9-h intervals. Terminal procedures were conducted 9–12 h post-final administration. Mouse characteristics are shown in Supplementary Table S4. For the hemolysis mouse model, six age-matched C57BL/6J males from the same supplier were subjected to two intraperitoneal injections of phenylhydrazine (PHZ) (60 mg/kg, Sigma-Aldrich, St. Louis, MO, USA) at 24-h intervals. Counterpart controls received equivalent volumes of saline. All specimens were maintained under identical housing conditions with ethical oversight as previously specified.

### 2.8. Immunofluorescence Staining and Hemoglobin Autofluorescence

Immunofluorescence staining was performed to analyze PS exposure on red blood cells (RBCs) and to identify liver sinusoidal endothelial cells (LSECs) in murine and human liver tissue. Murine RBCs were incubated with Annexin V Polyclonal Antibody (Proteintech, Rosemont, IL, USA, #11060-1-AP, 1:200) overnight at 4 °C. After three washes with PBS, cells were resuspended in Alexa Fluor 647-conjugated donkey anti-rabbit IgG (Dianova, Hamburg, Germany, #711605152, 1:500) for 1 h at room temperature. Cells were visualized using a fluorescence microscope (ZEISS Axio Scan.Z1, Carl Zeiss Microscopy GmbH, Jena, Germany).

Frozen liver sections from ethanol- and PHZ-treated mice, as well as cryopreserved human liver biopsies from patients with alcohol-related liver disease (ALD), were incubated overnight at 4 °C with CD206 antibody (Bio-Rad, Kidlington, UK, HS-488 003, 1:500) to detect LSECs. After PBS washes, slides were incubated with Alexa Fluor 647-conjugated donkey anti-rabbit IgG (Dianova, Hamburg, Germany; #711605152, 1:1000) for 1 h at room temperature in the dark. DAPI (Sigma-Aldrich, St. Louis, MO, USA) was used as a nuclear counterstain. Imaging was performed with the same fluorescence microscope.

Hemoglobin autofluorescence was visualized using the Alexa Fluor 488 channel, as described previously [8]. Autofluorescence displayed a distinct signal pattern comparable to conventional anti-hemoglobin antibody staining and reduced background from nonspecific antibody binding. Final imaging settings included 20× magnification. Although hemoglobin autofluorescence was detected in the green (488 nm) channel, it was rendered as red in merged images for intuitive contrast and clarity.

### 2.9. Histological Identification of LSECs

CD206 immunofluorescence was used to visualize the sinusoidal scavenger compartment. LSECs show a continuous linear distribution pattern of CD206 corresponding to the endothelial lining of the sinusoids. This pattern is distinctly different from the punctate distribution of macrophage markers (such as CD163) [8], supporting spatial interpretation. Therefore, CD206 expression was not used as an exclusive marker of liver sinusoidal endothelial cells, as the mannose receptor is also expressed by macrophages and other cell types. Cellular attribution was therefore based on anatomical localization and distribution pattern rather than marker expression alone.

### 2.10. Patients

Patients were enrolled from 2015 until 2021 at Salem Medical Center Heidelberg during an ongoing study. All patients were heavy drinkers (>80 g per day in males and >60 g per day in females) with ALD and a mean alcohol consumption of 187 +/− 124 g/day. The study protocol was reviewed and approved by the local Ethics Committee (S150-15) and conformed to the ethical guidelines of the 1975 Declaration of Helsinki, and all patients gave written informed consent inclusion. In n = 25 patients with liver biopsy, mRNA expression levels of Stabilin-1, Nrf2, and HO-1 were measured by q-PCR in liver tissue. Patient characteristics are shown in Supplementary Table S2. In further 8 patients with liver biopsy, immunostaining was performed in frozen liver sections. The patient characteristics are shown in Supplementary Table S5.

### 2.11. Statistical Analysis

All data are shown as mean ± standard deviation. Significant differences (n.s., not significant; *, *p* < 0.05; **, *p* < 0.01; ***, *p* < 0.001; ****, *p* < 0.0001) between datasets were assessed by one-way ANOVA with either Tukey’s or Dunnett’s test or an unpaired two-tailed Student’s *t*-test using GraphPad Prism 9 software. Correlations were analyzed using Spearman’s rank correlation coefficient. All experiments were performed in at least three independent biological replicates. Data shown represent technical replicates (n = 3) from a representative experiment. Individual data points were not displayed to maintain clarity, but variability across independent experiments was consistent.

## 3. Results

### 3.1. Evidence for Uptake of Oxidatively Damaged Red Blood Cells by Liver Sinusoidal Endothelial-like Cells

**Figure 1:**
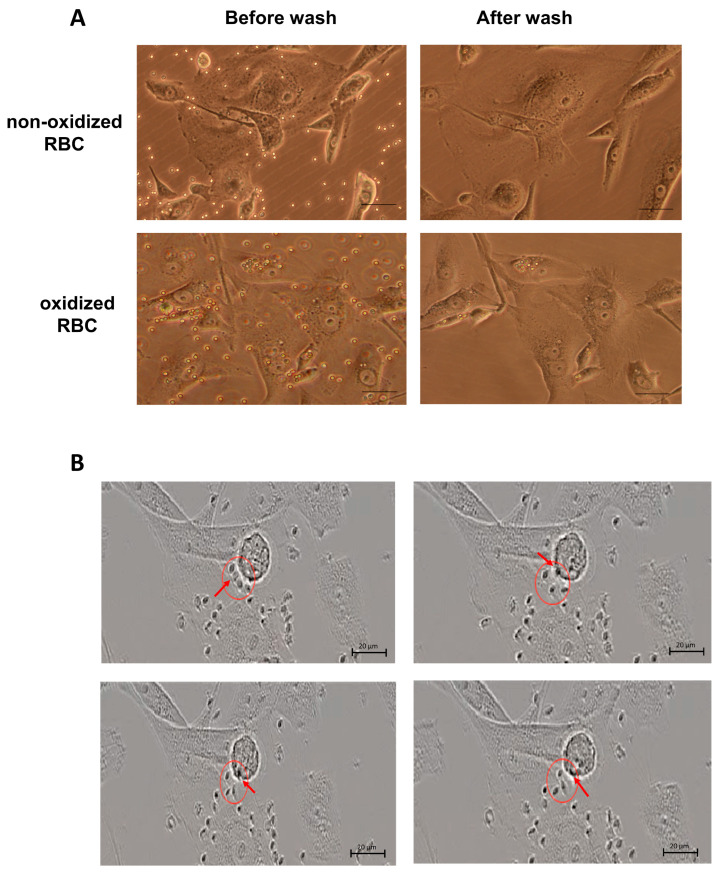
Direct evidence for red blood cell (RBC) efferocytosis by liver sinusoidal endothelial cells (LSECs). (**A**) Uptake of oxidized RBCs by SK-HEP1 cells. RBCs were oxidized using copper sulfate and (+)-sodium-L-ascorbate for 120 min and co-cultured with SK-HEP1 cells. After three PBS washes, oxidized RBCs remained intracellularly (**lower panels**), while non-oxidized RBCs were completely removed (**upper panels**). Scale bar: 20 µm. (**B**) Time-lapse imaging of RBC uptake. SK-HEP1 cells were co-cultured with 0.01% oxidized RBCs. Live cell imaging revealed a clear efferocytosis event occurring after ~22 h of incubation. Still frames taken over a 6-min interval illustrate the progressive uptake of a single RBC by an SK-HEP1 cell (red circles).

To investigate the capacity of LSECs to clear red blood cells (RBCs), we established an in vitro efferocytosis model using SK-HEP1 cells, an LSEC-like tumor-derived cell line. As shown in Figure 1A, prior to washing, both RBC types were observed on or near SK-HEP1 cells. However, after three gentle wash steps, only oxidized RBCs remained attached or internalized, while non-oxidized RBCs were almost completely removed. Some oxidized RBCs appeared aligned around the SK-HEP1 cell nuclei, indicating intracellular localization rather than surface adherence. To further confirm efferocytosis, live-cell imaging over 24 h demonstrated that oxidized RBCs were rapidly internalized by rounded SK-HEP1 cells (a morphology characteristic of post-mitotic rounding). With a temporal resolution of 2 min per frame, the uptake appeared to occur within this narrow time window (Figure 1B and Video S1). These findings provide experimental evidence that liver sinusoidal endothelial-like cells are capable of recognizing and internalizing oxidatively damaged red blood cells in vitro, consistent with an efferocytic phenotype.

### 3.2. Kinetic Regulation of HO-1 Expression by RBC Efferocytosis in SK-HEP1 Cells

**Figure 2:**
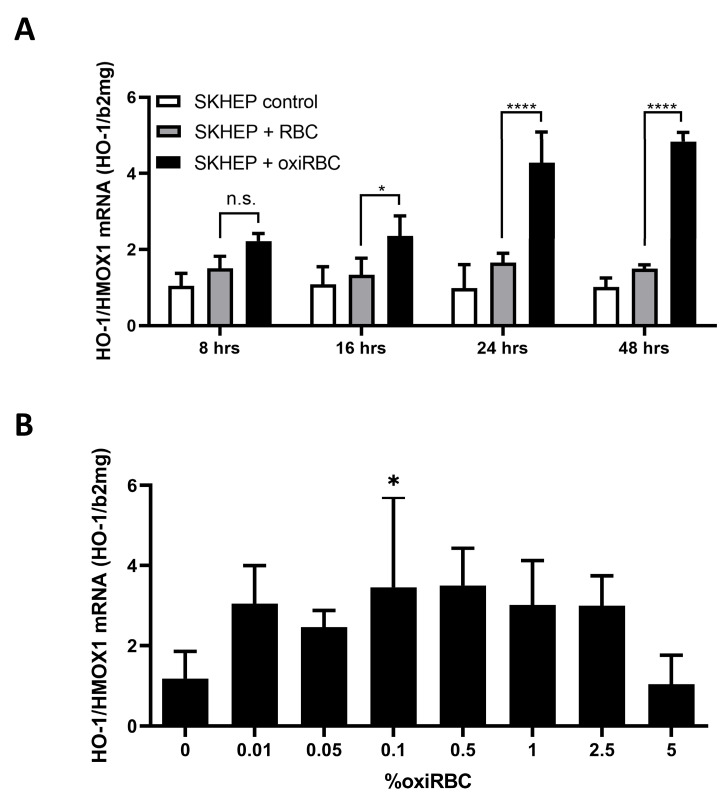
HO-1 induction and cytotoxicity in liver sinusoidal endothelial cells (LSECs) exposed to oxidized red blood cells (oxiRBCs). (**A**) Time-dependent induction of HO-1 mRNA. RBCs (0.5% hematocrit) were oxidized with CuSO_4_ for 2 h and co-cultured with SKHEP cells for varying time points. A significant upregulation of HO-1 mRNA was observed after 24 h, while non-oxidized RBCs had minimal effects. Expression was measured by qRT-PCR and normalized to β2-microglobulin (n = 3). *t*-test: n.s., not significant; *, *p* < 0.05; ****, *p* < 0.0001. (**B**) Dose-dependent induction of HO-1. SK-HEP1 cells were co-cultured with increasing concentrations of oxiRBCs. Both HO-1 and ferritin mRNA levels peaked at 0.5% hematocrit. Data are normalized to β2-microglobulin and presented as mean ± SD (n = 3). Dunnett’s test: *, *p* < 0.05.

We next examined the time- and dose-dependent effects of RBC exposure on HO-1 mRNA induction in SK-HEP1 cells. Time-course experiments showed that HO-1 mRNA levels began to rise at 8 h and peaked at 24 h following exposure to oxidized RBCs (~5-fold induction; Figure 2A). In contrast, HO-1 expression remained unchanged in cells co-cultured with non-oxidized RBCs, even after 48 h. Dose–response analysis revealed that HO-1 induction commenced at hematocrit concentrations as low as 0.01%, peaked at 0.5%, and progressively declined at higher RBC densities (Figure 2B). Interestingly, SK-HEP1 cell counts increased in parallel with RBC concentration up to 0.1% hematocrit but declined beyond this point (Supplementary Figure S1), cellular toxicity of oxiRBCs in SK-HEP1 cells under the same conditions is shown in Supplementary Figure S2, mirroring the HO-1 expression profile. These findings suggest that uptake or association of oxidized RBCs by LSEC-like cells is initiated at low RBC burden but becomes self-limiting at higher exposure levels, potentially due to cellular stress or saturation of uptake capacity.

### 3.3. Transcriptional Response of SK-HEP1 Cells to RBC Uptake: Involvement of Nrf2, Bmp6, and Ferritin

**Figure 3:**
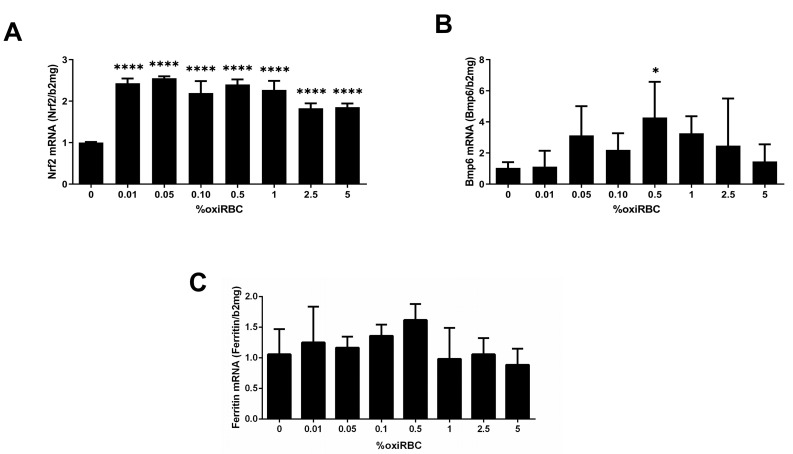
Induction of stress response and iron-regulatory genes during efferocytosis of oxidized red blood cells (oxiRBCs) by liver sinusoidal endothelial cells (LSECs). (**A**) Dose-dependent induction of Nrf2. SK-HEP1 cells were co-cultured with oxiRBCs at increasing hematocrit concentrations. Nrf2, a key transcriptional activator of HO-1, was significantly upregulated already at 0.05% hematocrit. (**B**) Bmp6 mRNA expression. Bmp6, a hepatokine involved in iron homeostasis, was likewise upregulated, peaking at 0.5% oxiRBC. (**C**) Ferritin mRNA expression. Ferritin, an iron-storage protein, showed a dose-dependent induction with peak expression at 0.5% hematocrit. mRNA levels were measured by quantitative real-time PCR and normalized to β2-microglobulin. Data are presented as mean ± SD (n = 3). Dunnett’s test: *, *p* < 0.05; ****, *p* < 0.0001.

We first examined key transcriptional regulators involved in the efferocytic response of SK-HEP1 cells to oxidized RBCs. Nrf2, a major upstream activator of HO-1 and redox-regulated detoxification genes [20], was induced early during co-culture, with maximal mRNA expression observed at 0.05% hematocrit (Figure 3). In contrast, HO-1 expression in Figure 2B peaked later, at 0.5% hematocrit, suggesting sequential transcriptional activation. BMP6, a downstream target of the Keap1–Nrf2–Bach1 axis involved in iron homeostasis, showed a similar induction pattern as HO-1, peaking at intermediate RBC concentrations and declining at higher levels. Likewise, ferritin expression followed the HO-1 response curve, with maximal expression at 0.5% hematocrit, indicating coordinated transcriptional regulation of iron-handling pathways during RBC efferocytosis. These findings indicate that oxidized RBC uptake by LSEC-like cells triggers a coordinated transcriptional program involving Nrf2 activation, heme degradation, and iron sequestration responses.

### 3.4. Stabilin-1 Mediates Phosphatidylserine-Dependent Efferocytosis of Oxidized RBCs

**Figure 4:**
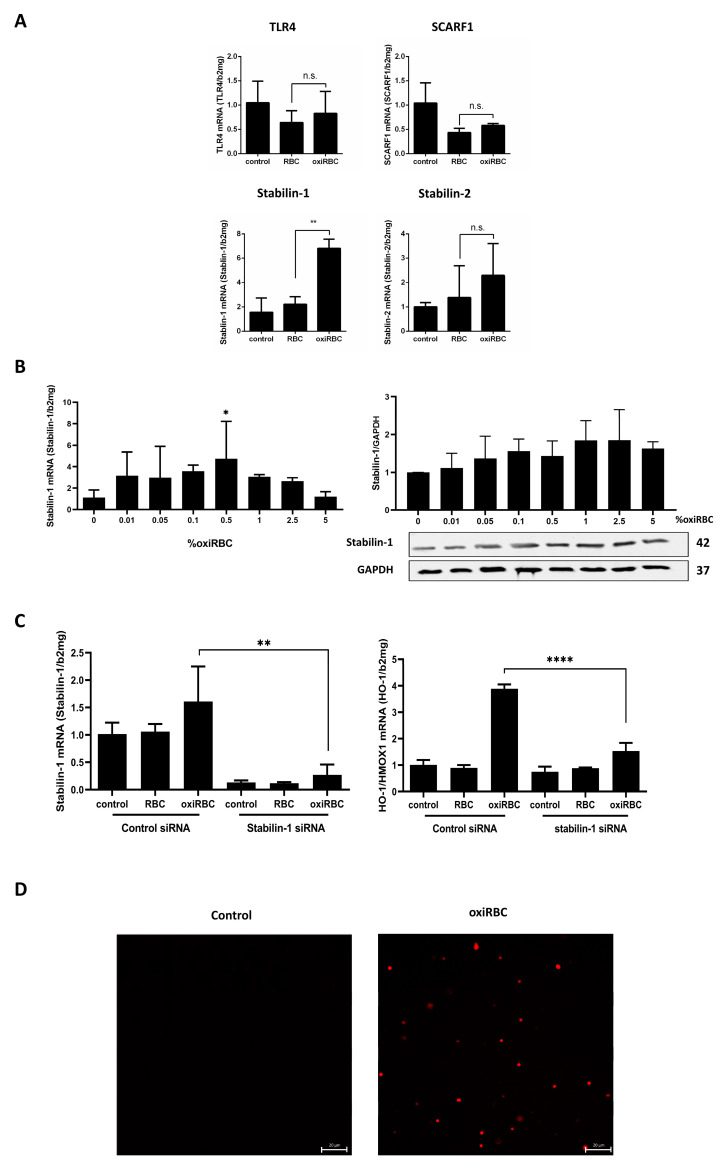
Stabilin-1 mediates recognition and uptake of oxidized red blood cells (oxiRBCs) by liver sinusoidal endothelial cells (LSECs). (**A**) Induction of scavenger receptors during efferocytosis. SK-HEP1 cells were co-cultured with 0.5% non-oxidized or oxidized RBCs for 24 h. Among several receptors tested (TLR4, ASGPR1, Stabilin-2), only Stabilin-1 mRNA was significantly upregulated in response to oxiRBCs. mRNA expression was quantified by qRT-PCR and normalized to β2-microglobulin (n = 3). Tukey’s test: n.s., not significant; **, *p* < 0.01. (**B**) Dose-dependent induction of Stabilin-1. Increasing concentrations of oxiRBCs induced Stabilin-1 mRNA expression (left panel) and protein expression (right panel) in a dose-dependent manner, with a peak at 0.5% hematocrit. RBC were oxidized by CuSO_4_ for 2 h and then SK-HEP1 cells were co-culture with different doses of oxidized RBC for 24 h (n = 3). Dunnett’s test: *, *p* < 0.05. (**C**) Functional role of Stabilin-1 in efferocytosis. SK-HEP1 cells were transfected with Stabilin-1 siRNA or control siRNA. Knockdown was confirmed after 48 h. Co-culture with 0.5% oxiRBCs resulted in ~60% reduction in HO-1 induction in Stabilin-1-silenced cells, supporting its role in oxiRBC uptake (n = 3). *t*-test: **, *p* < 0.01; ****, *p* < 0.0001. (**D**) Phosphatidylserine (PS) exposure on oxidized RBCs. Annexin V staining revealed increased PS externalization on oxiRBCs compared to controls, consistent with PS serving as an “eat-me” signal for Stabilin-1-mediated efferocytosis. Antibody: Annexin V Polyclonal (Proteintech, #11060-1-AP).

To identify the receptor responsible for RBC recognition by LSEC-like cells, we analyzed candidate scavenger receptors after exposure to oxidized RBCs. Among the tested genes (SCARF1, Stabilin-1, Stabilin-2, TLR4), only Stabilin-1 was significantly induced at both the mRNA and protein level (Figure 4A,B). Expression peaked at 0.5% hematocrit and declined at higher concentrations, mirroring HO-1 expression. Western blot confirmed that Stabilin-1 protein levels tracked with its mRNA expression (see also the original Western blot in Supplementary Figure S3). To directly assess its functional relevance, we performed siRNA-mediated knockdown of Stabilin-1, which reduced Stabilin-1 mRNA by >80% at 48 h. While control cells and non-oxidized RBCs showed no change in HO-1, cells co-cultured with oxidized RBCs exhibited a ~60% reduction in HO-1 expression following Stabilin-1 knockdown (Figure 4C), indicating impaired efferocytic signaling. Finally, we examined the “eat-me” signal presented by oxidized RBCs. Immunofluorescence staining revealed significantly increased PS exposure on oxidized vs. non-oxidized RBCs (Figure 4D). These findings suggest that Stabilin-1 recognizes PS on damaged RBCs, initiating their uptake by LSEC-like cells. Taken together, our mechanistic data suggest a PS-Stabilin-1 axis as a possible key trigger for uptake of oxidized RBCs by liver sinusoidal endothelial-like cells.

### 3.5. Ethanol Primes RBCs for Efferocytosis by Liver Sinusoidal Endothelial-like Cells

**Figure 5:**
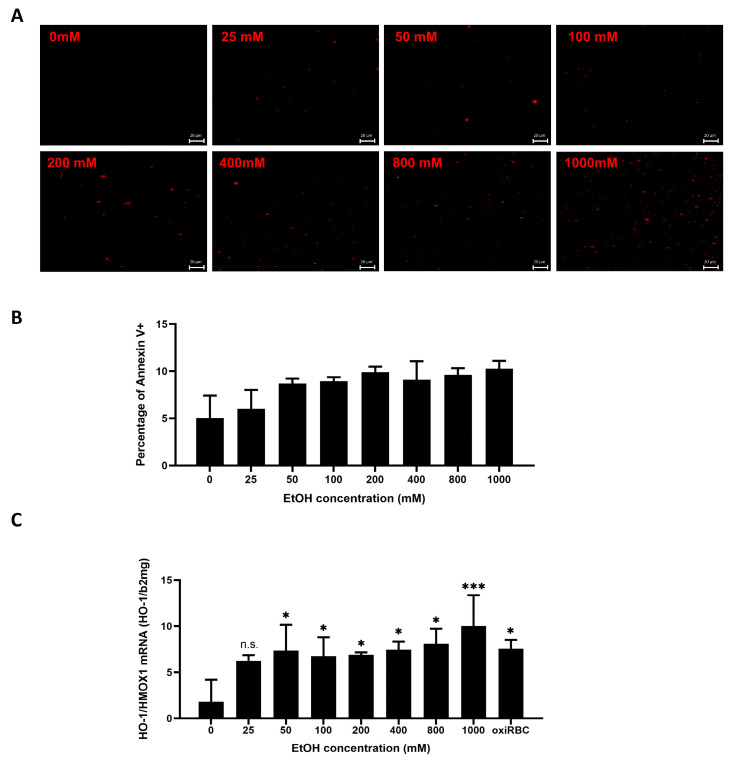
Ethanol primes red blood cells (RBCs) for liver sinusoidal endothelial cell (LSEC)-mediated efferocytosis via phosphatidylserine (PS) exposure. (**A**) Ethanol-induced PS externalization on RBCs. RBCs were treated with increasing concentrations of ethanol (25–1000 mM) for 24 h. Annexin V staining demonstrated progressive PS exposure on the outer membrane, detectable from ≥25 mM ethanol. (**B**) Quantification of PS exposure. Bar graph summarizing Annexin V-positive RBCs after ethanol treatment. Data are presented as percentage of Annexin V+ fluorescence (mean ± SD, n = 3). Dunnett’s test, not significant. (**C**) HO-1 induction as functional efferocytosis readout. SK-HEP1 cells were co-cultured with ethanol-treated RBCs (0.5% hematocrit) for 24 h. HO-1 mRNA expression, used as surrogate marker of efferocytic activity, increased in a dose-dependent manner starting from ≥25 mM ethanol, reaching levels comparable to copper-oxidized RBCs (positive control). Untreated RBCs served as negative control. Data normalized to β2-microglobulin ± SD (n = 3). Dunnett’s test: n.s., not significant; *, *p* < 0.05; ***, *p* < 0.001.

To simulate pathophysiological conditions relevant to alcohol-associated liver disease (ALD), we established an in vitro model in which RBCs were pretreated with increasing concentrations of ethanol for 24 h, followed by co-culture with SK-HEP1 cells. Copper sulfate–oxidized RBCs served as a positive control, and untreated RBCs as a negative control. As shown in Figure 5A, PS externalization—detected by Annexin V staining—increased progressively with rising ethanol concentrations. Notably, ethanol as low as 25 mM induced a significant proportion of Annexin V-positive RBCs (Figure 5B), indicating membrane oxidation and “eat-me” signal exposure. Correspondingly, HO-1 mRNA expression in SK-HEP1 cells was initiated at 25 mM ethanol and increased in a dose-dependent manner, reaching levels comparable to the oxidized RBC control at 400 mM ethanol (Figure 5C). These findings demonstrate that RBCs are highly sensitive to ethanol and undergo surface changes that promote their recognition and uptake by LSEC-like cells, even at physiologically relevant alcohol concentrations.

### 3.6. HO-1 Induction by Lysed RBCs and Hemin: Comparison with Oxidized Intact RBCs

**Figure 6:**
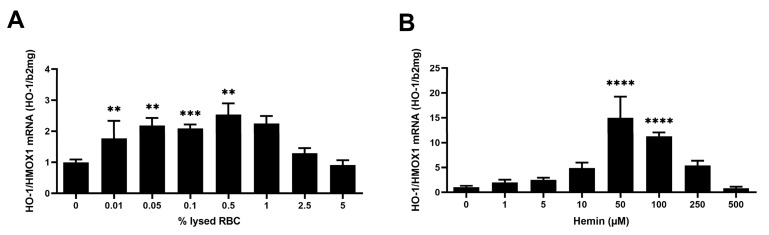
Differential induction of HO-1 by lysed red blood cells (RBCs) and free hemin in liver sinusoidal endothelial cells (LSECs). (**A**) Biphasic HO-1 response to RBC lysates and hemin. SK-HEP1 cells were co-cultured for 24 h with either lysed RBCs (0–5%) or corresponding concentrations of hemin (0–500 µM; calculated as 1% oxiRBC ≈ 100 µM hemin). Both stimuli induced a biphasic HO-1 mRNA response, with maximal expression at 0.5% lysed RBCs (~50 µM hemin) and at 50 µM hemin. Data are presented as mean ± SD (n = 3). Dunnett’s test: **, *p* < 0.01; ***, *p* < 0.001. (**B**) Stronger HO-1 induction by hemin. At peak concentrations, HO-1 mRNA expression was ~6.5-fold higher in hemin-treated cells than in cells exposed to lysed RBCs, indicating that free hemin acts as a more potent oxidative stimulus than RBC-derived debris. HO-1 mRNA levels were quantified by quantitative real-time PCR and normalized to β2-microglobulin. Data are presented as mean ± SD (n = 3). Dunnett’s test: ****, *p* < 0.0001.

Based on the calculated equivalence of 1% oxidized RBCs to approximately 100 μM hemin (Supplementary Table S3), we compared the HO-1 response of LSECs to lysed RBCs (0–5%) and corresponding concentrations of free hemin (0–500 μM). Both stressors elicited a biphasic HO-1 induction, peaking at 0.5% lysed RBCs and 50 μM hemin, respectively (Figure 6). However, at this peak concentration, hemin induced a 6.5-fold stronger HO-1 response compared to lysed RBCs, suggesting that free hemin imposes a higher oxidative burden on LSECs. In parallel, oxidized intact RBCs at 0.5% hematocrit induced a 1.5-fold higher HO-1 expression than lysed RBCs at the same RBC content, indicating that membrane-bound oxidation products may exert a more sustained efferocytic signal than cell debris.

### 3.7. Handling of RBC-Derived Material by LSECs In Vivo in Mouse Models of Hemolysis and Chronic Alcohol Exposure

**Figure 7:**
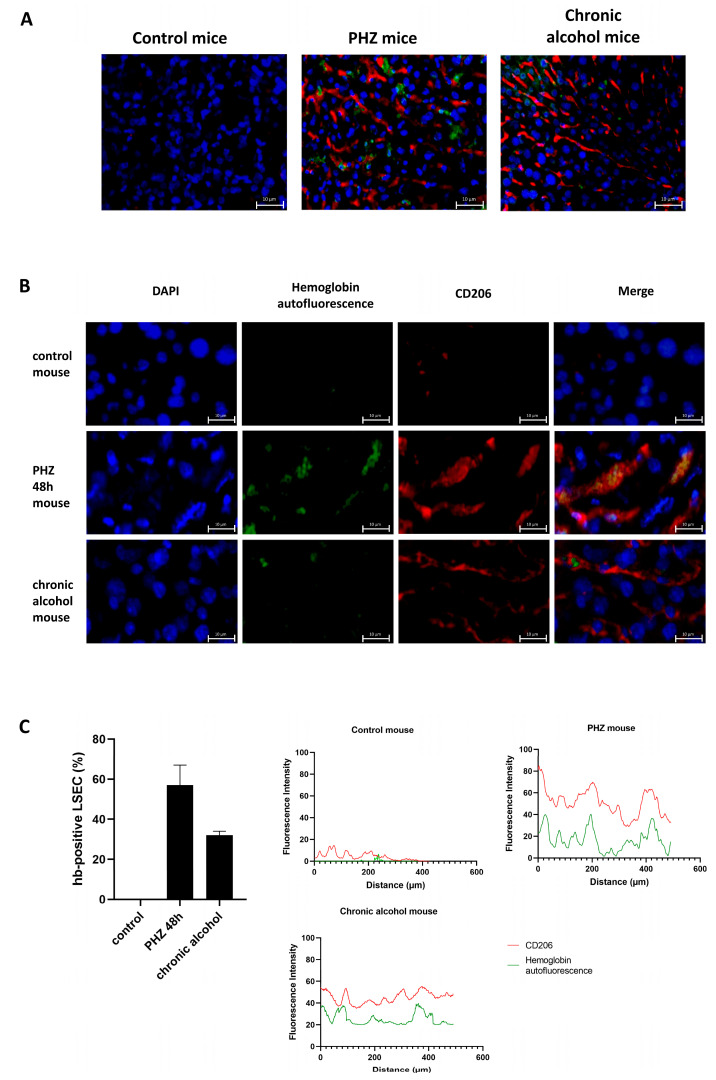
Efferocytosis by liver sinusoidal endothelial cells (LSECs) in murine models of hemolysis and chronic ethanol exposure. (**A**) Accumulation of RBC-derived hemoglobin signal. Hemoglobin autofluorescence (green) was markedly increased in liver sections from mice treated with phenylhydrazine (PHZ) or subjected to chronic ethanol feeding, compared to untreated controls. (**B**) Endothelial activation in response to erythrocyte turnover. CD206 staining (red), marking LSECs, was elevated in both PHZ- and ethanol-treated mice, indicating endothelial activation or expansion. Nuclei were counterstained with DAPI (blue). These findings support a role for LSECs in the hepatic clearance of damaged erythrocytes under conditions of both acute (PHZ) and chronic (ethanol) oxidative stress. (**C**) Quantification of percentage of hemoglobin (hb)-positive LSECs (autofluorescence) and fluorescence intensity distribution in different mouse models. Proportion of LSECs with positive hb-autofluoresence signal was counted manually and hb-autofluorescence and CD206 intensity distribution across the section was measured using ImageJ. (n = 3 per model).

To validate LSEC-mediated efferocytosis in vivo, we analyzed hemoglobin autofluorescence and CD206 expression in liver sections from control mice, phenylhydrazine (PHZ)-treated mice, and animals subjected to chronic ethanol feeding (Figure 7A,B). Figure 7A shows a low-magnification overview for comparison, channel-specific images are shown in Figure 7B. In healthy controls, hemoglobin autofluorescence (green) was nearly absent. PHZ-treated mice displayed strong hemoglobin-derived signals within CD206-positive sinusoidal structures displaying a continuous endothelial-like distribution pattern, consistent with localization to the sinusoidal endothelial scavenger compartment although CD206 is not specific for LSECs and conventional H&E staining does not allow reliable detection of erythrocyte-derived signals within sinusoidal structures. Mice exposed to chronic alcohol showed moderate hemoglobin accumulation in similar CD206-positive sinusoidal regions, distinct from the punctate staining pattern characteristic of Kupffer cells. A quantification of the proportion of hb-positive LSECs and the fluorescence signals is shown in Figure 7C. Notably, CD206 expression (a marker for LSECs) was lowest in control animals, intermediate after PHZ, and highest in alcohol-fed mice. This suggests differential LSEC responses depending on the duration and nature of oxidative stress: acute hemolysis induces transient RBC uptake without substantial endothelial remodeling, whereas chronic alcohol exposure appears to drive compensatory expansion of the LSEC compartment. These findings support the concept that LSECs are involved in handling RBC-derived materialin RBC clearance under both acute and chronic hemolytic conditions in vivo.

### 3.8. Hepatic Stabilin-1 Expression Mirrors Erythrophagocytic Stress in Heavy Drinkers

To investigate the clinical relevance of RBC clearance and endothelial responses in alcohol-associated liver disease (ALD), we analyzed a cohort of 47 heavy drinkers (mean daily intake: 187 g, age: 52.7 years, 66% male; Supplementary Table S2). Laboratory data revealed elevated transaminases (AST: 132 U/L, ALT: 71 U/L) and markedly increased GGT (509 U/L), consistent with alcohol-related liver injury. Signs of systemic inflammation (CRP: 12.7 mg/L) and macrocytic anemia (MCV: 95.3 fL) were present, alongside elevated ferritin (818 ng/mL), suggestive of iron overload and hemolysis. The mean serum sCD163 level (1210 ng/mL) exceeded the normal range, indicating increased macrophage scavenger activity. Elastography showed a mean liver stiffness of 21.5 kPa, and 18% of patients had sonographic signs of cirrhosis. Steatosis and increased CAP values (300 dB/m) were also common. To gain mechanistic insight, hepatic mRNA levels of stabilin-1 were measured in 25 biopsy-confirmed ALD patients from the Heidelberg biobank (Table 1). Stabilin-1 expression correlated strongly with ASGPR1 (r = 0.62, *p* = 0.001) and with Nrf2 (r = 0.56, *p* = 0.004), the upstream regulator of oxidative stress responses. A moderate correlation with HO-1 mRNA (r = 0.35, *p* = 0.097) supports the hypothesis that stabilin-1 is linked to the heme degradation and antioxidant pathways in ALD. Inverse correlations were observed with pigmented macrophage infiltration (r = −0.60, *p* = 0.005), lobular inflammation (r = −0.39, *p* = 0.087), and serum AST (r = −0.40, *p* = 0.053), suggesting that stabilin-1 upregulation may reflect a compensatory endothelial mechanism that mitigates inflammation and hepatocellular damage. Together, these findings suggest that hepatic stabilin-1 expression mirrors oxidative and erythrophagocytic stress in heavy drinkers and may serve as a functional marker of endothelial adaptation in ALD.

### 3.9. Hemoglobin Uptake by LSECs in Heavy Drinkers with and Without Clinical Signs of Hemolysis

**Figure 8:**
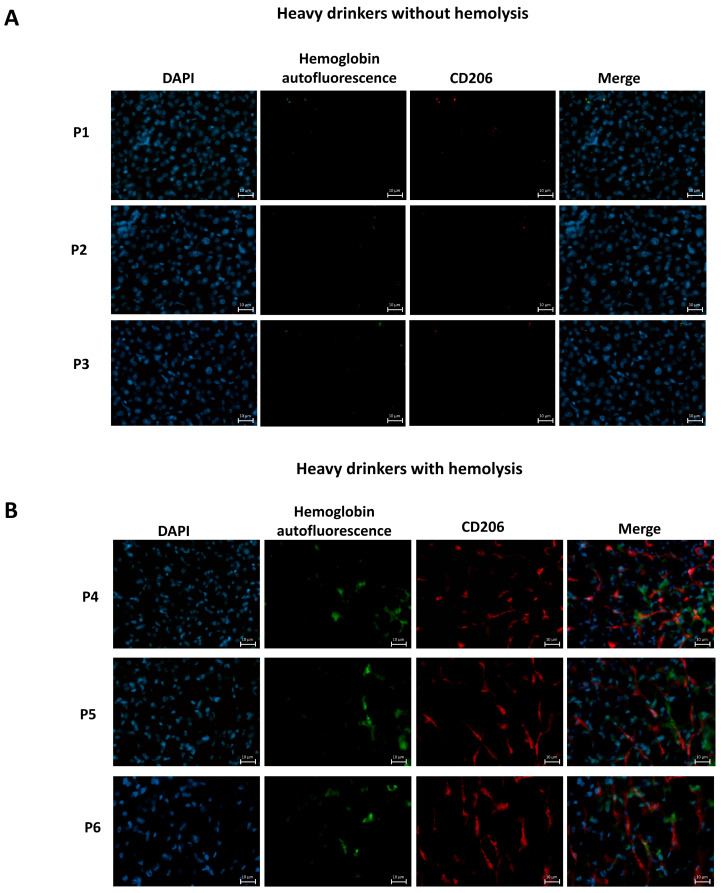
Erythrophagocytosis by liver sinusoidal endothelial cells (LSECs) in human alcoholic liver disease (ALD) with and without hemolysis. (**A**) Minimal LSEC activation in non-hemolytic ALD. Immunofluorescence staining of cryopreserved liver tissue from ALD patients without clinical signs of hemolysis (P1–P3) revealed low hemoglobin autofluorescence (green) and sparse CD206-positive LSECs (red). Nuclei were counterstained with DAPI (blue). (**B**) Enhanced LSEC-mediated clearance in hemolytic ALD. Liver sections from hemolytic ALD patients (P4–P6), defined by anemia, hyperferritinemia, and elevated sCD163, showed pronounced hemoglobin autofluorescence co-localizing with CD206+ LSECs consistent with increased erythrocyte-derived signal accumulation.

To assess whether findings from mouse models of hemolysis translate to human alcohol-related liver disease (ALD), we performed immunofluorescence staining on cryopreserved liver sections from patients with ALD, comparing individuals with and without clinical evidence of hemolysis. Hemoglobin autofluorescence (green), CD206-positive liver sinusoidal endothelial cells (LSECs, red), and nuclear DAPI staining (blue) were visualized in merged images (Figure 8A,B). In non-hemolytic patients (Figure 8A), hemoglobin autofluorescence was minimal and largely absent from CD206-positive regions, suggesting a lack of endothelial hemoglobin uptake. In contrast, liver sections from patients with clinical and laboratory signs of hemolysis, such as low hemoglobin, elevated ferritin, and increased soluble sCD163, showed strong hemoglobin autofluorescence co-localizing with CD206-positive LSECs (Figure 8B). These patients also exhibited cirrhotic architecture and elevated liver stiffness. Importantly, hemoglobin autofluorescence was not restricted to endothelial structures but also observed in hepatocytic and non-endothelial regions. This is consistent with previous findings from our group demonstrating that both hepatocytes [29] and hepatic macrophages [8] are capable of erythrocyte uptake in ALD. The present study, however, specifically focused on LSECs, whose role in erythrophagocytosis has been largely overlooked to date. Areas devoid of staining (appearing as signal gaps) correspond histologically to lipid droplets, as confirmed by hematoxylin and eosin (HE) staining in adjacent sections. The presence of hemoglobin-derived signals within CD206-positive sinusoidal regions supports involvement of the sinusoidal endothelial scavenger compartment in handling hemolysis-derived material in human alcohol-related liver disease. However, definitive discrimination between intact erythrocyte uptake and intracellular hemoglobin deposition cannot be achieved without ultrastructural analyses. Although it remains technically challenging to distinguish between intact erythrocytes and processed hemoglobin remnants in situ, the observed staining patterns align with a model in which LSECs contribute to intrahepatic detoxification of hemolysis-derived heme and iron in advanced alcohol-related liver disease.

The number of CD206+ cells was increased in hemolytic cirrhotic samples, suggesting activation or expansion of the LSEC compartment in response to ongoing red blood cell turnover. Autofluorescence signals were also detected in hepatocytes and non-endothelial regions, consistent with prior reports of erythrophagocytosis by other hepatic cell types. Signal-free areas corresponded to lipid droplets, as confirmed by H&E staining.

### 3.10. Colocalization Validates Hemoglobin Autofluorescence in Human Cirrhotic Liver

**Figure 9:**
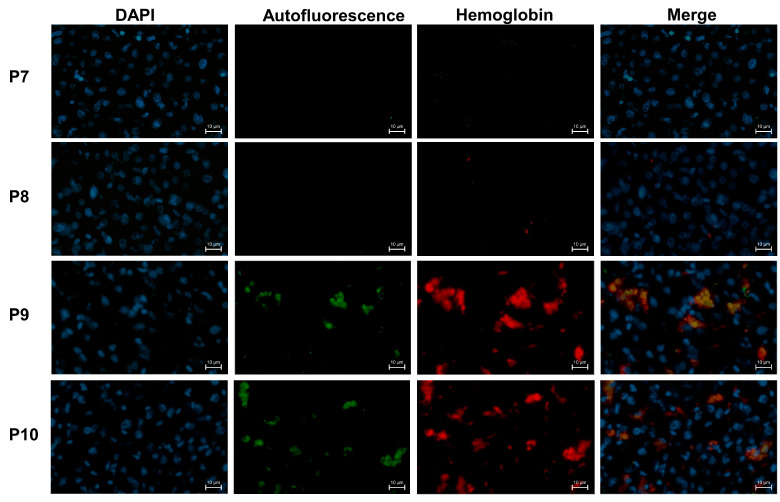
Colocalization confirms hemoglobin autofluorescence in human alcoholic liver disease (ALD) with and without hemolysis. In liver sections from patients with hemolytic ALD (P9, P10), the anti-hemoglobin antibody signal (647 nm, red) strongly colocalized with the autofluorescence signal in the 488 nm channel (green), confirming that the autofluorescence originated from hemoglobin. In contrast, liver tissue from non-hemolytic ALD patients (P7, P8) showed only baseline autofluorescence in the 488 nm channel and no detectable anti-hemoglobin staining. These results indicate that hemoglobin-derived autofluorescence is a specific pathological hallmark of hemolytic ALD.

To validate that the observed autofluorescence (channel 488) in frozen liver sections from patients with hemolytic ALD indeed represents hemoglobin, we performed additional immunofluorescence staining using an anti-hemoglobin antibody (channel 647, Product # PA5-145321, Thermo Fisher Scientific, 20 µg/mL). Antibody signals showed strong colocalization with the autofluorescence, confirming that the autofluorescence predominantly originates from hemoglobin (Figure 9). Notably, non-hemolytic patients exhibited lower CD206 expression, consistent with the findings shown in Figure 8. Clinical characteristics of the analyzed patients are summarized in Supplementary Table S6.

## 4. Discussion

This study provides mechanistic in vitro evidence and anatomically consistent in vivo observations that the liver sinusoidal endothelial scavenger compartment participates in the handling of oxidatively damaged red blood cell-derived material in alcohol-related liver disease. While LSECs have long been recognized for their endocytic and scavenger functions, including expression of receptors such as Stabilin-1 and -2 [41,42], their role in efferocytosis of red blood cells remained unexplored. We now suggest a novel RBC–LSEC axis that complements the known macrophage-mediated clearance via CD163 [2] and reveals a distinct, endothelial-driven pathway in ALD.

Using live-cell imaging, we observed rapid uptake of oxidized RBCs by SK-HEP1 cells, a surrogate for human LSECs. Uptake occurred within minutes, particularly in rounded mitotic cells, consistent with prior reports linking efferocytosis to cytoskeletal changes during cell division [43,44]. In vivo, we provide indirect evidence for RBC uptake by LSECs in PHZ-treated mouse livers. While previous studies described increased hepatic RBC sequestration under hemolytic or inflammatory conditions [31,33], we provide morphological evidence consistent with endothelial RBC internalization.

Mechanistically, oxidized RBCs induced strong HO-1 and Nrf2 activation in SK-HEP1 cells. Both genes responded dose-dependently, peaking at intermediate hematocrit and declining at higher levels, suggesting a toxicity threshold limiting efferocytosis. RBC exposure also triggered BMP6 and ferritin, indicating activation of antioxidant and heme-catabolic pathways [20]. Out of all tested receptors, only Stabilin-1 was consistently upregulated. Silencing Stabilin-1 reduced HO-1 induction by ~60%, supporting its role as an efferocytic receptor in LSECs and extending its known function in apoptotic and acetylated LDL clearance [42] to include RBC turnover.

We further show that ethanol primes RBCs for efferocytosis via PS externalization, a known “eat-me” signal [45]. Immunofluorescence confirmed increased PS exposure on ethanol-treated RBCs, and SK-HEP1 cells co-cultured with these RBCs upregulated HO-1 in a dose-dependent manner. These data align with previous reports of ethanol-induced eryptosis [46] and establish a mechanistic link between alcohol and endothelial RBC clearance.

Comparative assays showed that hemin triggered stronger HO-1 induction than lysed RBCs, yet intact oxidized RBCs were more potent than lysates, highlighting the role of membrane-associated signals in efferocytosis. In alcohol-fed mice, we observed increased Stabilin-1-positive LSECs, suggesting adaptive remodeling in response to ongoing RBC stress. In human ALD samples, hepatic Stabilin-1 expression correlated with Nrf2 and ASGPR1, but not with classical inflammatory markers, underscoring a distinct endothelial pathway [2].

Importantly, our patient data are consistent with these experimental findings. In human cirrhotic livers, hemoglobin autofluorescence co-localized with CD206-positive LSECs in patients showing clinical/lab signs of hemolysis (low Hb, high ferritin, sCD163), but not in those without hemolysis. Autofluorescence also appeared in hepatocytes and macrophages [8,29], supporting a multicellular clearance response, with LSECs as an underappreciated contributor. Lipid-rich zones lacked autofluorescence, consistent with histological steatosis. To validate that the autofluorescence indeed reflects hemoglobin, we performed co-staining with an anti-hemoglobin antibody. In hemolytic ALD patients, antibody signals strongly co-localized with the 488 nm autofluorescence channel, whereas non-hemolytic patients showed only baseline autofluorescence and no detectable antibody staining. This strongly suggests that hemoglobin-derived autofluorescence is a specific hallmark of hemolytic ALD and rules out unspecific background signals.

Our data support an ethanol-triggered efferocytic pathway in LSECs mediated by Stabilin-1 and potentially CD206. Stabilin-1, a scavenger receptor highly expressed in LSECs, co-localized with RBC fragments and was upregulated in both mouse and human ALD samples. CD206 (mannose receptor) is a C-type lectin expressed by macrophages, immature dendritic cells, and liver sinusoidal endothelial cells, where it functions as an endocytic receptor involved in glycoprotein and ligand clearance [47]. In the liver, CD206 expression within sinusoidal endothelial cells is characterized by a diffuse, continuous distribution along the sinusoidal network, in contrast to the punctate, cell-restricted staining pattern of Kupffer cells. Accordingly, in the present study CD206 was interpreted as a marker of the sinusoidal scavenger compartment rather than as an exclusive endothelial cell marker. To our knowledge, neither receptor has been directly linked to erythrophagocytosis before. Recognition of hemoglobin adducts, PS patches, or alcohol-induced glycan changes may represent a novel clearance paradigm [48].

Whether alcohol concentrations capable of inducing phosphatidylserine (PS) exposure occur in vivo is a relevant question. While the mean blood alcohol concentration (BAC) in our patient cohort was ~0.1% (17 mM), systemic BAC values reflect rapid equilibration and do not capture transient local exposures during alcohol intake. As previously discussed by us [8], ethanol concentrations in the upper gastrointestinal lumen can transiently approach the concentration of the ingested beverage (typically 10–40% *v*/*v*) before dilution and systemic mixing. Because ethanol readily diffuses across biological membranes, erythrocytes passing the splanchnic circulation may be transiently exposed to higher ethanol levels than those measured in peripheral blood. Although such short-term erythrocyte exposure cannot be quantified in vivo, this concept provides a biologically plausible framework for erythrocyte priming during drinking. In line with this model, we observed PS externalization already at 25 mM ethanol, indicating that relatively low ethanol concentrations are sufficient to induce erythrocyte membrane alterations that promote LSEC-mediated clearance. This mechanistic framework is consistent with epidemiological links between binge drinking patterns and the development of alcoholic hepatitis [49,50,51].

A notable finding was robust BMP6 induction in SKHEP1 cells exposed to oxidized RBCs. We previously showed that endothelial BMP6 is essential for hepatocellular hepcidin induction [36]. Our current data identify RBC uptake—not heme alone—as a potent trigger of BMP6 expression, suggesting that hemolysis and RBC turnover directly activate endothelial iron-sensing pathways [20,52].

Hemolysis is increasingly recognized as a driver of morbidity in ALD [2,8]. We show that not only macrophages and hepatocytes [8,29], but also LSECs, internalize RBC-derived material, evidenced by hemoglobin autofluorescence in ALD liver tissue. Historical descriptions of stomatocytosis and Zieve syndrome [53,54] and modern data on eryptosis [55] support the concept of a clearance-prone erythrocyte phenotype in alcoholics. Resulting intrahepatic heme and iron accumulation may exacerbate oxidative stress, hyperbilirubinemia, and ferroptosis—a form of iron-dependent cell death relevant in ALD [7,56].

Several limitations must be acknowledged. SK-HEP1 cells do not represent bona fide LSECs but a surrogate model with endothelial and tumor-derived features. For example, SK-HEP1 fenestrations have a smaller diameter (55 ± 28 nm) and lower porosity (2.0 ± 1.4%), mostly scattered individually with only rudimentary sieve plates. Primary LSECs have fenestrations of 50–200 nm diameter, often clustered into typical sieve plates, with higher porosity. After VEGF stimulation, SK-HEP1 pore diameter increases to 104 ± 59 nm, resembling the dynamic response of LSECs, but overall, they are less dense and organized than primary cells [57,58]. SK-HEP1 cells also demonstrate extensive uptake of ligands, such as Human Plasma low density lipoprotein (Dil-Ac-LDL) [58] and formaldehyde serum albumin (FITC-albumin) [38]. SK-HEP-1 cells also inherently express scavenger receptors, including Stabilin-2 (STAB2), MRC1 (CD206), and PECAM-1 (CD31) at both mRNA and protein levels [59] while primary LSECs have a much greater capacity for endocytosis and degradation than SK-HEP1 cell models [60,61]. In addition, validation of these findings in primary LSECs will be required in future studies. Direct quantification of RBC uptake following Stabilin-1 knockdown was also not performed and remains a limitation of the study. Finally, extended biochemical and inflammatory markers (e.g., ALT, AST, GGT) were not systematically assessed in all experiments, which limits the comprehensive characterization of the in vivo alcohol model. CD206 is not cell-type specific and was therefore interpreted in the context of anatomical localization and sinusoidal distribution rather than marker exclusivity. Analyses of human liver tissue rely on hemoglobin autofluorescence and immunofluorescence, which do not allow unequivocal discrimination between intact erythrocyte uptake and intracellular hemoglobin deposition. In addition, functional blocking experiments and ultrastructural analyses were not performed and will be required for definitive mechanistic conclusions.

While this study establishes the involvement of the Stabilin-1–PS axis, further work is needed to validate this interaction under physiological conditions and to elucidate downstream signaling events. In human cirrhotic liver tissue, elevated Stabilin-1 expression may also reflect endothelial activation or remodeling associated with advanced fibrosis and should therefore not be interpreted as a direct surrogate of increased phagocytic activity.

## 5. Conclusions

In summary, we provide evidence consistent with a previously unrecognized efferocytosis mechanism in endothelial-like cells, triggered by ethanol-primed red blood cell injury and mediated by phosphatidylserine recognition most likely via Stabilin-1. This pathway activates antioxidant and iron-handling programs and may contribute to hepatic iron overload and sinusoidal dysfunction in ALD. While erythrophagocytosis by Kupffer cells and hepatocytes is well established [8,29], our findings establish LSECs as an additional key component of a multicellular hepatic red blood cell clearance network, supported by patient tissue data. Further studies are warranted to define the long-term consequences of LSEC-mediated erythrocyte clearance and its therapeutic implications.

## Figures and Tables

**Table 1:** Spearman correlations of Stabilin-1 and Nrf2 with HO-1 mRNA in liver tissue from patients with alcoholic liver disease (ALD). Spearman rank correlation analysis was performed in N = 28 ALD patients with available liver biopsy samples. Stabilin-1 mRNA expression showed a moderate, non-significant correlation with HO-1 mRNA levels (ρ = 0.35, *p* = 0.09), while Nrf2 expression correlated strongly and significantly with HO-1 (ρ = 0.56, *p* = 0.004).

		Stabilin-1/b2mg (mRNA)	
Parameter	Category	r	*p*
ASGPR1/b2mg	mRNA	0.62	0.001
Nrf2/b2mg	mRNA	0.56	0.004
AST (U/L)	Laboratory	−0.40	0.053
Pigmented macrophages 0–1 ^1^	Histology	−0.60	0.005
M65 (U/L) ^2^	Special laboratory	−0.45	0.068
Albumin/b2mg	mRNA	0.36	0.082
Lobular inflammation 0–3 ^3^	Histology	−0.39	0.087
HO1/b2mg	mRNA	0.35	0.097

^1^: histological score of presence of pigmented macrophages by Prussian Blue stain, ^2^: M65: serum level of cytokeratin 18, ^3^: histological presecence lobular inflammation from Kleiner score.

## Data Availability

The raw data supporting the conclusions of this article will be made available by the authors on request.

## Supplementary Materials

The following supporting information can be downloaded at: https://www.mdpi.com/article/10.3390/biology15090699/s1, Table S1: Primer sequences used for quantitative real-time PCR (qPCR) and (B) antibody for immunofluorescence, Table S2: Clinical characteristics of heavy-drinking patients included in hepatic Stabilin-1 expression analysis, Table S3: Calculation of hemin equivalents corresponding to lysed red blood cell (RBC) percentages, Table S4: Mouse characteristics, Table S5: Clinical characteristics of heavy-drinking patients included in LSECs immunofluorescence analyses, Table S6: Clinical characteristics of heavy-drinking patients included in hemoglobin immunofluorescence analyses, Figure S1: Cytotoxic effects of oxidized RBCs on SK-HEP1 cells, Figure S2: Independent replicate confirming oxiRBC-induced toxicity in SK-HEP1 cells, Figure S3. Original stabilin-1 Western blot for Figure 4B; Video S1. SKHEP Video.

## Abbreviations

The following abbreviations are used in this manuscript:

ALD	alcohol-related liver disease
ASGPR1	asialoglycoprotein receptor 1
b2mg	Beta2-microglobulin
BAC	blood alcohol concentration
BMP6	bone morphogenetic protein 6
GGT	gamma-glutamyltransferase
GST	glutathione S transferase
Hp	haptoglobin
HPx	hemopexin
HO-1	heme oxygenase 1
HTC	hematocrit
LSECs	liver sinusoidal endothelial cells
M65	serum levels of cytokeratin 18
MCV	mean corpuscular volume of erythrocytes
Nrf2	nuclear factor erythroid 2-related factor 2
oxiRBC	oxidized RBC
PS	phosphatidylserin
RBC	red blood cell
