# Supplementary material for: Phosphatidylserine-Dependent Clearance of Damaged Red Blood Cells by Liver Sinusoidal Endothelial Cells in Alcohol-Related Liver Disease
Source: Biology (Basel). 2026 Apr 29;15(9):699. doi: 10.3390/biology15090699 (PMC13163024; doi:10.3390/biology15090699)
Supplement: Supplementary file 1 [file biology-15-00699-s001.zip › Supplementary Tables and Figures.pdf]

Supplementary Material

Supplementary tables

Suppl. Table S1: (A) Primer sequences used for quantitative real-time PCR (qPCR) and (B) antibody for immunofluorescence

List of forward and reverse primer sequences and antibody used for gene expression analysis in human and murine samples.

A

Gene		Sequence
**Human**		
HO-1	Forward	5'-CCA GGC AGA GAA TGC TGA GTT C-3'
	Reverse	5'- AAG ACT GGG CTC TCC TTG TTG C-3'
Beta-2-microglobulin (b2mg)	Forward	5’-TGA CTT TGT CAC AGC CAA AGA TA-3'
	Reverse	5’-AAT CCA AAT GCG GCA TCT TC-3'
Ferritin	Forward	5'-TAC GAG CGT CTC CTG AAG ATG C-3'
	Reverse	5'-GGT TCA GCT TTT TCT CCA GGG C-3'
Nrf2	Forward	5'-CAC ATC CAG TCA GAA ACC AGT GG-3'
	Reverse	5'-GGA ATG TCT GCG CCA AAA GCT G-3'
ASGPR1	Forward	5'-GAA GCA GTT CGT GTC TGA CCT G-3'
	Reverse	5'-AGC GAG AGA ACC AGT AGC AGC T-3'
ASGPR2	Forward	5'-AGA ACG CAC ACC TGG TGG TCA T-3'
	Reverse	5'-TTC CAA GAG CCA TCA CTG TCC G-3'
SCARF1	Forward	5'-AGC TAC CGT GTC CAG GAT GAA G-3'
	Reverse	5'-GGC TCG ATG AAG CTG TGG TTG A-3'
TLR4	Forward	5’-CCC TGA GGC ATT TAG GCA GCT A-3'
	Reverse	5’-AGG TAG AGA GGT GGC TTA GGC T-3'
Bmp6	Forward	5’-GTC GTA ATC GCT CTA CCC AGT CC-3'
	Reverse	5’-CTG GGT AAT AAG GCA CTG GCA TG -3'

B

Antigen	Host species	Dilution	Stock concentration (mg/mL)	Company	Cat. No.
**CD206**	rabbit	1:500	0.5	HistoSure	HS-488 003
**GAPDH**	rabbit	1:3000		CST	2118S
**Annexin V**	rabbit	1:200	2.0	Proteintech	11060-1-AP
**Rabbit IgG**	Donkey Alexa Fluor® 647-conjugated	1:500		jacksonimmuno.com	711-605-152
**Hemoglobin**	rabbit	1:250	0.5	Thermo Fisher Scientific	PA5-145321

Suppl. Table S2: Clinical characteristics of heavy-drinking patients included in hepatic Stabilin-1 expression analysis

Summary of demographic and laboratory parameters of patients whose liver tissue was used for Stabilin-1 mRNA quantification.

**Patient characteristics (N=47)**			
	**Normal range**	**Mean**	**SD**
**General information**			
Sex (male)		66%	
Age (years)		52.7	13.0
BMI (kg/m^2)	18-25	25.5	5.1
Alcohol consumption (g/day)	<40 (m), <20 (f)	187	124
**Routine laboratory**			
AST (U/L)	<50	132	145
ALT (U/L)	<50	71	66
GGT (U/L)	<60	509	726
AP (U/L)	40-130 (m), 35-105 (f)	113	65
Bilirubin total (mg/dL)	<1.3	1.7	2.3
INR	0.95-1.10	1.04	0.39
Hemoglobin (g/dL)	13.5-17.5 (m), 12-16 (f)	13.6	2.0
Hematocrit (%)	40-53 (m), 36-48 (f)	38.4	5.4
MCV (fL)	80-96	95.3	7.1
Erythrocytes (/pL)	4.5-5.9 (m), 4.1-5.1 (f)	4.1	0.7
Leukocytes (/nL)	3.7-10.0 (m), 3.5-10.0 (f)	8.3	3.4
Platelets (/nL)	150-360	184.2	74.0
Ferritin (ng/mL)	30-400 (m), 13-150 (f)	818	779
CRP (mg/L)	<0.5	12.7	31.5
Albumin (g/dL)	3.8-5.9	4.1	0.7
Transferrin saturation (%)	16-45	43.0	25.1
Serum iron (µg/dL)	59-158 (m), 37-145 (f)	125.2	68.8
Triglycerides (mg/dL)	<150	162.1	145.3
LDH (U/L)	<250	254	82
CD163 (ng/mL)	<800	1210	813
**Ultrasound**			
Liver size (cm)	<16	16.4	4.4
Hepatic steatosis (0-3)	0	1.9	0.8
Spleen size (cm)	<11.5	10.1	1.9
Ascites (1 or 0)	0	13%	
Signs of cirrhosis (1 or 0)	0	18%	
**Transient elastography**			
Liver stiffness (kPa)	<6	21.5	26.7
CAP (dB/m)	<240	300	48

Suppl. Table S3: Calculation of hemin equivalents corresponding to lysed red blood cell (RBC) percentages

Approximate conversion of lysed RBC percentages into micromolar hemin concentrations used in *in vitro* stimulation assays

Calculation of hemin equivalents from lysed RBCs

The hemoglobin concentration in whole blood was assumed to be 130 g/L (within the normal range of 110–150 g/L in females). Accordingly, 250 µL whole blood contains 32,500 µg hemoglobin, which is assumed to be fully retained within the packed RBC fraction after centrifugation (200 µL).

Thus, 10 µL packed RBCs contain 32,500 µg × (10 / 200) = 1,625 µg Hb.

1% (v/v) RBC = 10 µL RBCs in a total volume of 1 mL

Hb molecular weight: 67,000 g/mol
Mass (g) = Concentration (M) × Volume (L) × Molecular weight (g/mol)

Hb concentration of 1% RBCs (µM) =
1,625 (µg) / 0.001 (L) / 67,000 (µg/µmol) = 24.25 µM

Globin accounts for ~96%, heme for ~4%
1,625 µg Hb contains:
1,625 × 4% = 65 µg hemin

Hemin molecular weight: 616.5 g/mol
Hemin concentration of 1% RBCs (µM) =
65 (µg) / 0.001 (L) / 616.5 (µg/µmol) = 105.44 µM

Conclusion: 1% RBC corresponds to 24.25 µM Hb and 105.44 µM hemin

Suppl. Table S4: Mouse characteristics

	Controls		Chronic EtOH model		PHZ model	
**Parameter**	**Mean**	**SD**	**Mean**	**SD**	**Mean**	**SD**
**Leukocytes ($\times 10^3$ /µL)**	43	20	26	14	24	4
**Erythrocytes ($\times 10^6$/µL)**	9.2	0.9	9.7	0.6	3.9	0.3
**Hemoglobin (g/dL)**	15.4	1.1	16.4	1.4	7.8	0.4
**Hematocrit (%)**	45.1	3.5	47.4	3.7	19.8	1.5
**LDH (U/L)**	424.3	363.1	1045.0	791.1	4433.0	671.8
**AST (U/L)**	193.0	133.4	583.8	459.9	237.5	17.7
**ALT (U/L)**	38.8	13.4	127.2	67.8	36.5	9.2
**Serum iron (µmol/L)**	37.0	28.0	20.8	6.0	26.9	5.9
**Albumin (g/L)**	24.5	12.7	23.7	6.1	23.7	1.6

Suppl. Table S5: Clinical characteristics of heavy-drinking patients included in LSECs immunofluorescence analyses

Demographic, clinical, and laboratory features of patients whose liver cryosections were used for immunofluorescent staining of RBC-derived hemoglobin and CD206-positive LSECs.

			Heavy drinkers without hemolysis			Heavy drinkers with hemolysis		
**Parameter**	**Units**	**Category**	**P1**	**P2**	**P3**	**P4**	**P5**	**P6**
**Sex**	male: 1	General	0	1	1	0	0	1
**Age**	years	General	50	42.2	41	70.8	44.4	58.6
**BMI**	Kg/m²	Morphometric	26.3	26.1	27.8	20.4	23.7	21.3
**AST**	U/L	Laboratory	33	31	43	177	285	175
**ALT**	U/L	Laboratory	32	39	71	95	66	49
**GGT**	U/L	Laboratory	51	78	291	841	3533	822
**AP**	U/L	Laboratory	58	71	85	135	371	127
**Bilirubin total**	mg/dL	Laboratory	0.4	0.3	0.2	2	8.3	2.5
**Hemoglobin**	g/dL	Laboratory	15.1	15.5	16.7	9.6	12.7	13.4
**MCV**	fL	Laboratory	87	86	88	114	112	105
**Erythrocytes**	/pL	Laboratory	4.9	5.2	5.0	2.4	3.3	3.6
**Ferritin**	ng/mL	Laboratory	132	96	147	2420	1887	1573
**CRP**	mg/L	Laboratory	0.5	0.5	3.8	1	67.7	61.8
**Liver size**	cm	Ultrasound	18.4	16.8	17	15.5	40	20
**Hepatic steatosis**	0-3	Ultrasound	2	0	0	2	2	3
**Spleen size**	cm	Ultrasound	8.3		11	8.6	9.5	13.9
**Ascites**	1 or 0	Ultrasound	0	0	0	0		1
**Signs of cirrhosis**	1 or 0	Ultrasound	0	0	0	0		1
**Liver stiffness**	kPa	Ultrasound	2.5	6.5	4.3	12.2	70.6	73.5
**Kleiner score**	0-4	Histology	0	2	1	1	4	4

Suppl. Table S6: Clinical characteristics of heavy-drinking patients included in hemoglobin immunofluorescence analyses

Demographic, clinical, and laboratory features of patients whose liver cryosections were used for immunofluorescent staining of autofluorescence and hemoglobin antibody.

			Heavy drinkers without hemolysis		Heavy drinkers with hemolysis	
**Parameter**	**Units**	**Category**	**P7**	**P8**	**P9**	**P10**
**Sex**	male: 1	General	0	1	1	0
**Age**	years	General	50	42.2	49	59
**BMI**	Kg/m²	Morphometric	26.3	26.1	23.7	18.1
**AST**	U/L	Laboratory	33	31	271	104
**ALT**	U/L	Laboratory	32	39	149	88
**GGT**	U/L	Laboratory	51	78	391	241
**AP**	U/L	Laboratory	58	71	56	195
**Bilirubin total**	mg/dL	Laboratory	0.4	0.3	0.8	9.2
**Hemoglobin**	g/dL	Laboratory	15.1	15.5	13.7	12.1
**MCV**	fL	Laboratory	87	86	112	99
**Erythrocytes**	/pL	Laboratory	4.9	5.2	3.5	3.4
**Ferritin**	ng/mL	Laboratory	132	96	1126	1110
**CRP**	mg/L	Laboratory	0.5	0.5	2.27	56.86
**Liver size**	cm	Ultrasound	18.4	16.8	17.8	12
**Hepatic steatosis**	0-3	Ultrasound	2	0	3	
**Spleen size**	cm	Ultrasound	8.3			11
**Ascites**	1 or 0	Ultrasound	0	0	0	1
**Signs of cirrhosis**	1 or 0	Ultrasound	0	0	1	1
**Liver stiffness**	kPa	Ultrasound	2.5	6.5	28.4	59
**Kleiner score**	0-4	Histology	0	2	4	3

Supplementary figures

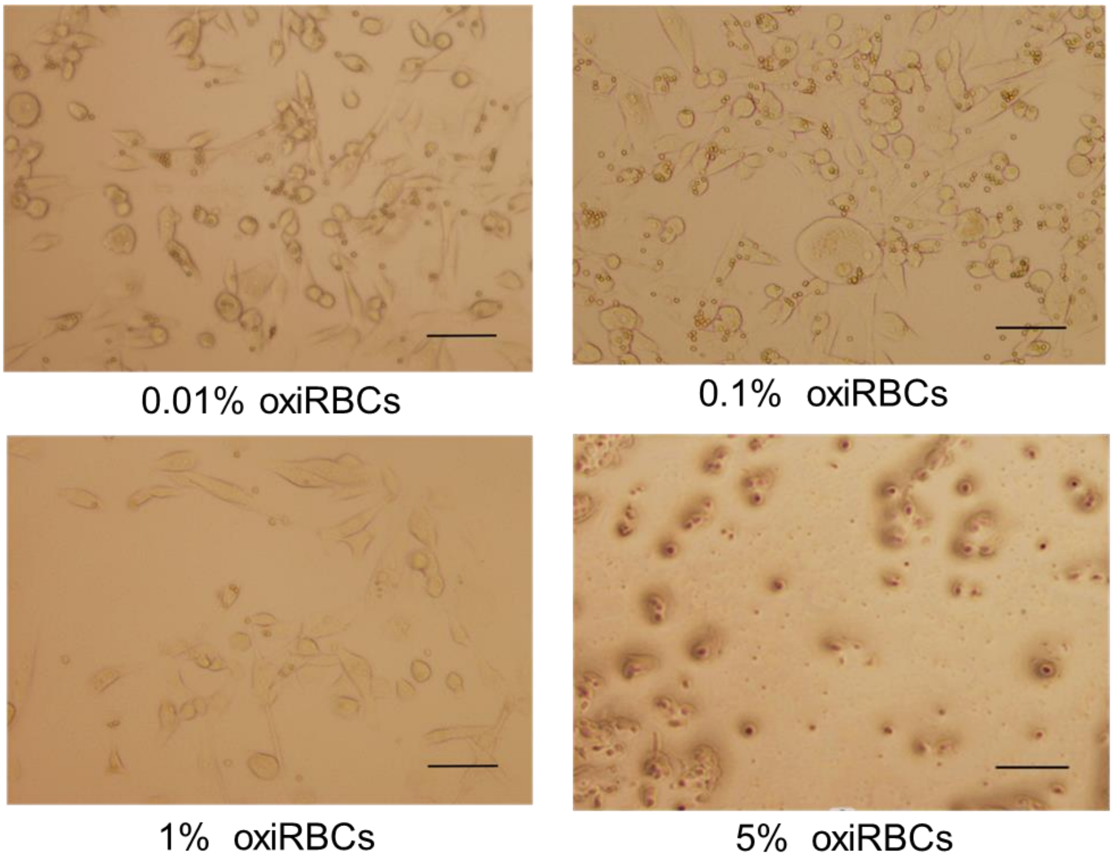

Supplementary Figure S1: Cytotoxic effects of oxidized RBCs on SK-HEP1 cells. SK-HEP1 cells were exposed to increasing concentrations of oxiRBCs for 24 hours. Cell density was visibly reduced at concentrations exceeding 0.5%, consistent with cytotoxic effects. Scale bar: 100 µm.

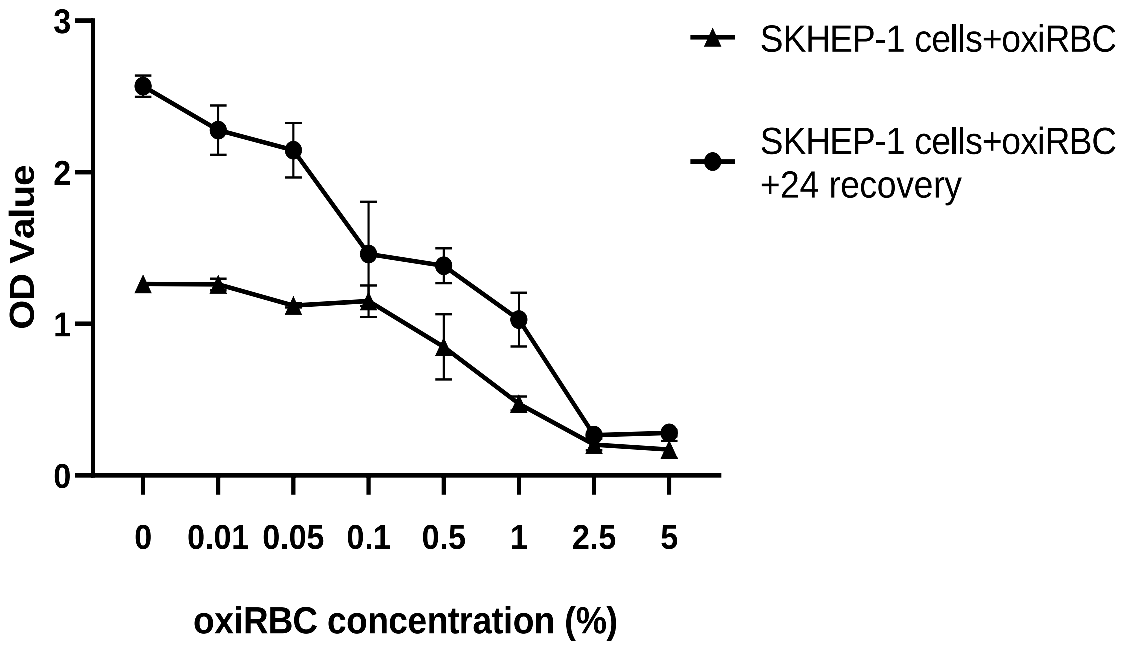

Supplementary Figure S2: Independent replicate confirming oxiRBC-induced toxicity in SK-HEP1 cells.

An independent biological replicate under identical conditions confirmed the concentration-dependent cytotoxicity of oxiRBCs, as shown in Supplementary Figure 1.

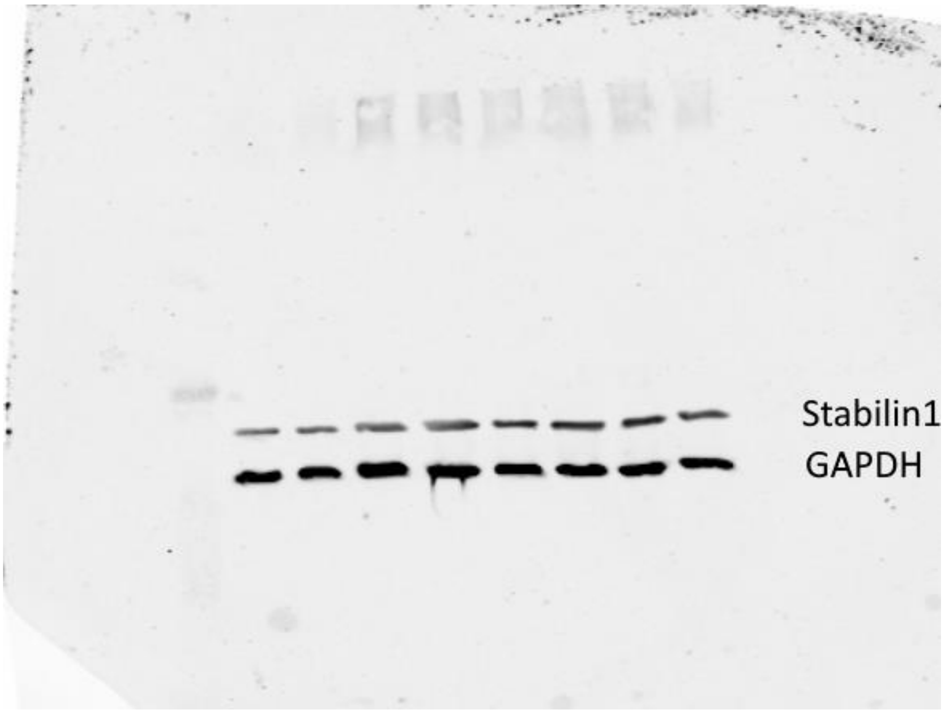

Supplementary Figure S3: Original stabilin-1 Western blot for Figure 4B.
